# GZ17-6.02 interacts with proteasome inhibitors to kill multiple myeloma cells

**DOI:** 10.18632/oncotarget.28558

**Published:** 2024-03-05

**Authors:** Laurence Booth, Jane L. Roberts, Cameron West, Paul Dent

**Affiliations:** ^1^Department of Biochemistry and Molecular Biology, Virginia Commonwealth University, Richmond, VA 23298, USA; ^2^Genzada Pharmaceuticals, Hutchinson, KS 67502, USA

**Keywords:** autophagy, ER stress, GZ17-6.02, bortezomib, proteasome inhibitor

## Abstract

GZ17-6.02, a synthetically manufactured compound containing isovanillin, harmine and curcumin, has undergone phase I evaluation in patients with solid tumors (NCT03775525) with a recommended phase 2 dose (RP2D) of 375 mg PO BID. GZ17-6.02 was more efficacious as a single agent at killing multiple myeloma cells than had previously been observed in solid tumor cell types. GZ17-6.02 interacted with proteasome inhibitors in a greater than additive fashion to kill myeloma cells and alone it killed inhibitor-resistant cells to a similar extent. The drug combination of GZ17-6.02 and bortezomib activated ATM, the AMPK and PERK and inactivated ULK1, mTORC1, eIF2α, NFκB and the Hippo pathway. The combination increased ATG13 S318 phosphorylation and the expression of Beclin1, ATG5, BAK and BIM, and reduced the levels of BCL-XL and MCL1. GZ17-6.02 interacted with bortezomib to enhance autophagosome formation and autophagic flux, and knock down of ATM, AMPKα, ULK1, Beclin1 or ATG5 significantly reduced both autophagy and tumor cell killing. Knock down of BAK and BIM significantly reduced tumor cell killing. The expression of HDACs1/2/3 was significantly reduced beyond that previously observed in solid tumor cells and required autophagy. This was associated with increased acetylation and methylation of histone H3. Combined knock down of HDACs1/2/3 caused activation of ATM and the AMPK and caused inactivation of ULK1, mTORC1, NFκB and the Hippo pathway. HDAC knock down also enhanced ATG13 phosphorylation, increased BAK levels and reduced those of BCL-XL. Collectively, our present studies support performing additional *in vivo* studies with multiple myeloma cells.

## INTRODUCTION

Multiple myeloma (MM) is a clonal disorder of plasma cells accounting for 13% of hematologic malignancies; ~35,000 patients per annum are diagnosed in the USA, with the disease more common in males [1–3]. Despite advances in the treatment of MM over the past 10–15 years with the introduction of proteasome inhibitors, e.g., bortezomib (Velcade, BTZ) and subsequently carfilzomib (Kyprolis) and ixazomib (Ninlaro), and more recently with immunomodulatory approaches and antibodies, the median life expectancy of MM patients remains sub-optimal at ~7–8 years [4–6]. However, not all myeloma patients respond to proteasome inhibitors and myeloma cells can evolve under this selective proteasome inhibitor pressure to become drug-resistant.

More recently, the myeloma therapeutics field has been dramatically altered by the use of CAR-T cells and other immunotherapeutics. [7–10]. Two CAR-T cells products, directed against the Anti-B-cell maturation antigen (BCMA), idecabtagene vicleucel (ide-cel) and ciltacabtagene autoleucel (cilta-cel), have recently received FDA approval for relapsed/refractory MM in patients who had already underwent four or more prior lines of therapy. Response rates of up to ~90% have been observed in patients who were otherwise untreatable. However, it has also been noted that CAR T-cell therapies are often associated with immune system adverse events, for example, cytokine release syndrome, various cytopenias, and infections. Some patients treated with CAR-T cells can also relapse after treatment. Collectively, this implies that there remains a patient population that could benefit from novel therapeutic approaches using GZ17-6.02.

The novel therapeutic agent GZ17-6.02 (602) is comprised of three synthetically manufactured natural compounds in the following ratio: isovanillin (77%), harmine (13%) and curcumin (10%) [11]. Curcumin as a single agent has low solubility in water, has very poor PK/PD *in vivo* and failed in the clinic as an anti-cancer agent [12, 13]. In our prior *in vitro* studies using low physiologic concentrations of curcumin, the generation of reactive oxygen species played an important role in the process by which tumor cells were killed [14]. However, the anti-tumor biology of curcumin when combined with isovanillin and harmine is different to that of free curcumin, apparently requiring minimal, if any, ROS generation [15–23].

Harmine is isolated from the plants *Arum palaestinum* and *Peganum harmala* and like curcumin, has been used as a medicinal herb for millennia [24–26]. Prior work has argued that harmine selectively kills tumor cells over normal tissues. Harmine can cause DNA damage and has been reported to inhibit drug efflux pumps. Isovanillin is an isomer of vanillin, isolated from the vanilla bean, and is an inhibitor of aldehyde oxidase and xanthine oxidase. It can donate a proton forming a hydrogen bond and accept three hydrogen bonds from other compounds [27–29]. Combined with our curcumin findings, we believe that isovanillin can complex with curcumin and harmine to create an entity with unique biology when compared to the three individual agents [14].

GZ17-6.02 received its IND in 2018 from the FDA and has undergone phase I evaluation in patients with solid tumors (NCT03775525). The RP2D is 375 mg PO BID. In the trial a PR was observed in a c-MET amplified NSCLC, a prolonged SD with 20% tumor shrinkage in a HER2 mutant NSCLC, and prolonged SD responses in multiple other tumor types, including the almost untreatable disease, uveal melanoma. The safety profile of the drug was outwardly benign in patients with only grade 1/2/3 reversible alterations in plasma liver enzyme levels. Laboratory-based PK/PD studies with the drug have shown that all three components of GZ17-6.02 were concentrated in tumors at concentrations above those used for our *in vitro* studies. In colorectal and prostate tumors GZ17-6.02 as a single agent significantly prolonged animal survival beyond the drug-treatment time-frame. We believe that developing GZ17-6.02 as a novel MM agent potentially opens up a multitude of novel opportunities to develop therapeutic approaches which will prolong patient survival.

## RESULTS

**Figure 1:**
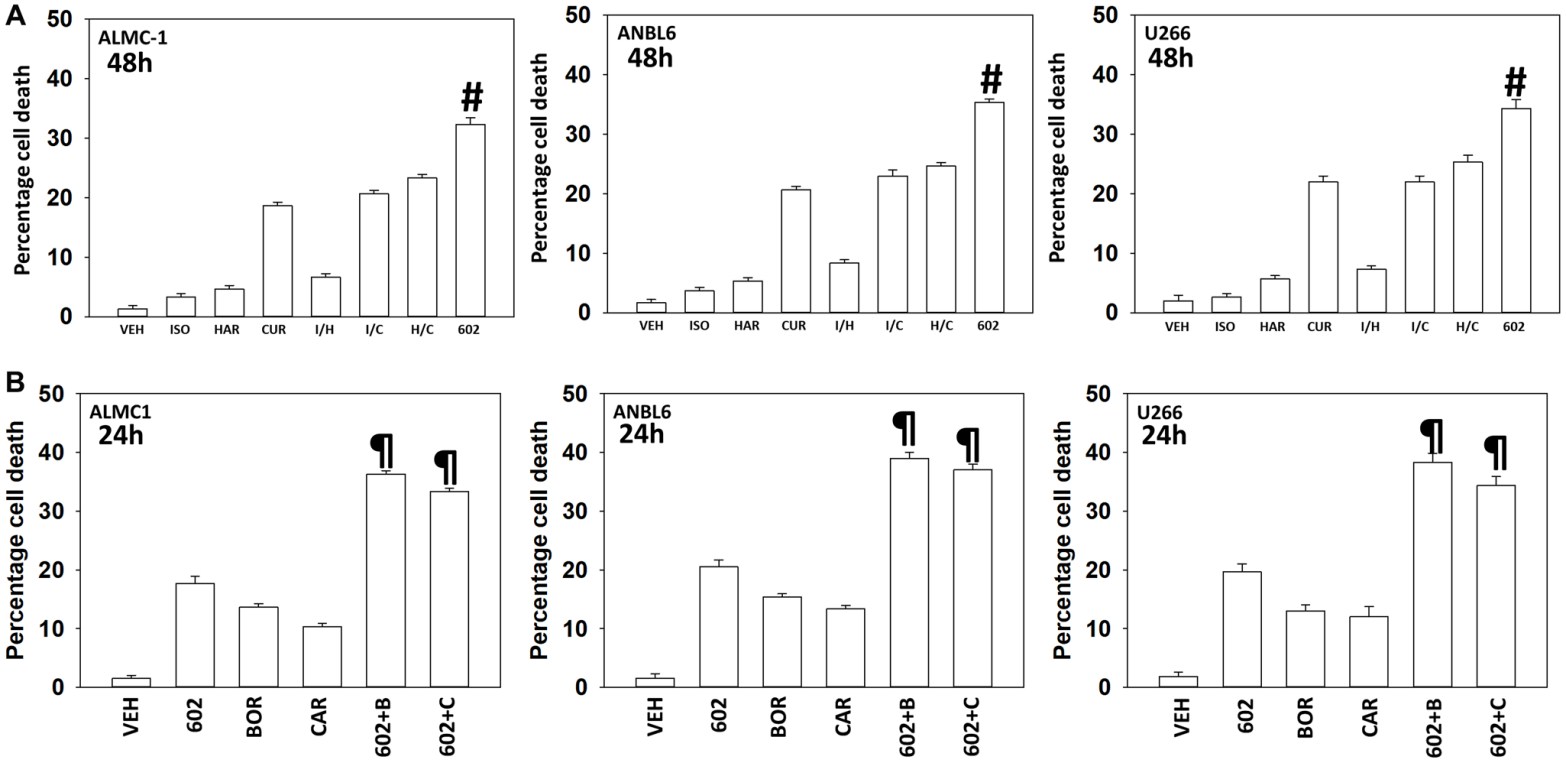
GZ17-6.02 and proteasome inhibitors interact in a greater than additive fashion to kill multiple myeloma cells. (**A**) ALMC1, ANBL6 and U266 cells were treated with vehicle control, GZ17-6.02 (curcumin (2.0 μM) + harmine (4.5 μM) + isovanillin (37.2 μM)) or with component parts of GZ17-6.02 as individual agents at the indicated concentrations or in duo combinations. Cells were isolated 48 h afterwards and viability determined via trypan blue exclusion assays (*n* = 3 +/− SD). ^#^ *p* < 0.05 greater than other tested drug treatments. (**B**) ALMC1, ANBL6 and U266 cells were treated with vehicle control, GZ17-6.02 (2 μM curcumin, final), bortezomib (10 nM), carfilzomib (5 nM) or the drugs in combination, as indicated. Twenty-four h later, cells were isolated, and viability determined via trypan blue exclusion assays (*n* = 3 +/− SD). ^¶^ *p* < 0.05 greater than other tested drug treatments.

Our first series of studies defined the interactions of the individual components of GZ17-6.02 in multiple myeloma cells. As single agents, only curcumin caused substantial significant cell killing 48 h after drug exposure (Figure 1A). The dual combination of harmine and isovanillin exhibited almost no further activity compared to harmine alone, which was similar to the combinations of curcumin with either agent to curcumin alone. In contrast, GZ17-6.02 which contains all three agents caused significantly greater levels of tumor cell killing than would be predicted based on the effects of the three individual components [14]. We next determined whether GZ17-6.02 interacted with proteasome inhibitors to kill MM cells. GZ17-6.02 interacted in a greater-than-additive fashion with both bortezomib and carfilzomib to kill MM cells 24 h after drug exposure (Figure 1B).

The data presented in Figure 1B with GZ17-6.02 as a single agent piqued our interest as we were observing ~20% tumor cell killing, which appeared to be higher than we previously reported in solid tumor cells. Hence, we made direct comparisons between the lethality of GZ17-6.02 in MM cells, prostate cancer cells and NSCLC cells. GZ17-6.02 was significantly more efficacious at killing MM cells when compared to prostate and lung cancer cells (Supplementary Figure 1). This is also of particular translational interest as we recently demonstrated that GZ17-6.02 as a single agent significantly prolonged survival in a mouse model of prostate cancer [17].

**Figure 2:**
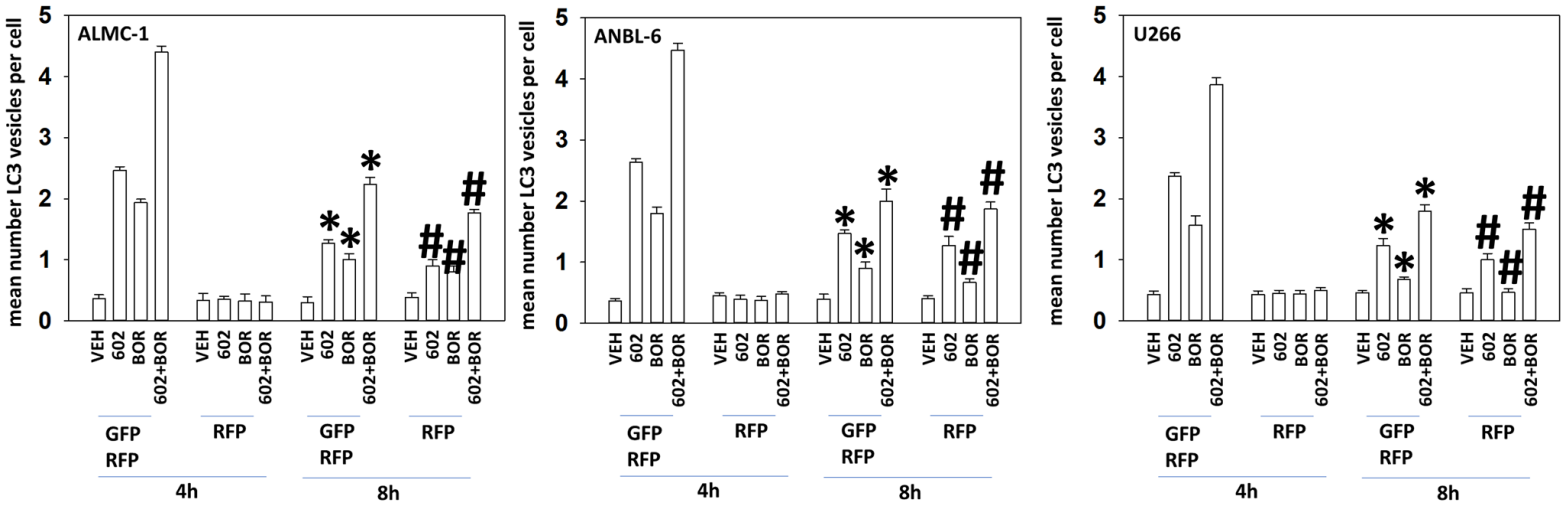
GZ17-6.02 and bortezomib interact to cause autophagosome formation and autophagic flux. ALMC1, ANBL6 and U266 cells were transfected with a plasmid to express LC3-GFP-RFP. Twenty-four h later, cells were treated with vehicle control, GZ17-6.02 (curcumin 2 μM, final), bortezomib (10 nM) or the drugs in combination for 4 h and 8 h. At each time point, the mean number of autophagosomes (GFP+ RFP+) and autolysosomes (RFP+) per cell were determined randomly in >100 cells. (*n* = 3 +/− SD). ^*^ *p* < 0.05 less than corresponding value at 4 h; ^#^ *p* < 0.05 greater than corresponding value at 4 h.

**Figure 3:**
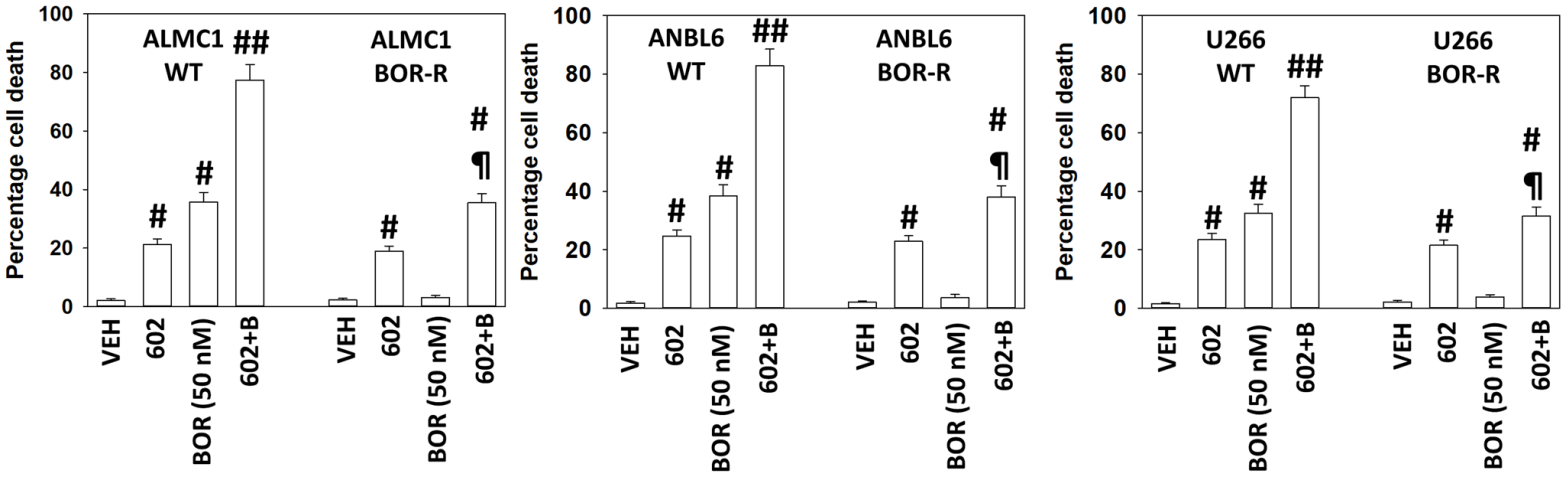
GZ17-6.02 efficacy is not significantly reduced in bortezomib-resistant multiple myeloma cells. Multiple myeloma cells were grown in 50 nM bortezomib for a month. Surviving “resistant” cells and parental cells were then treated with vehicle control, GZ17-6.02 (2 μM curcumin, final), bortezomib (50 nM) or the drugs in combination for 24 h. Cells were isolated after 24 h and viability determined by trypan blue exclusion assay (*n* = 3 +/− SD) ^#^ *p* < 0.05 greater than vehicle control; ^##^ *p* < 0.05 greater than GZ17-6.02 single agent value; ^¶^ *p* < 0.05 less than corresponding value in wild type parental cells.

A key mechanism mediating GZ17-6.02 lethality in solid tumor cells is by enhancing macroautophagy [15–23]. Both GZ17-6.02 and bortezomib enhanced the formation of autophagosomes in MM cells and interacted together to promote additional vesicle formation (Figure 2). Over time, the number of autophagosomes declined and the numbers of autolysosomes increased, indicative that we were also observing autophagic flux. In an identical fashion to our comparative studies in Supplementary Figure 1, we compared the ability of GZ17-6.02 to increase the formation of autophagosomes and autolysosomes in both liquid and solid tumor cell types (Supplementary Figure 2). GZ17-6.02 was significantly more capable of increasing autophagosome levels in MM cells than in prostate cancer cells. When we determined the subsequent formation of autolysosomes, however, there was no significant obvious difference between the two tumor cell types. MM cells made resistant to BTZ were killed by GZ17-6.02 in a similar manner to parental sensitive cells, however, the interaction between GZ17-6.02 and BTZ was considerably reduced (Figure 3).

**Figure 4:**
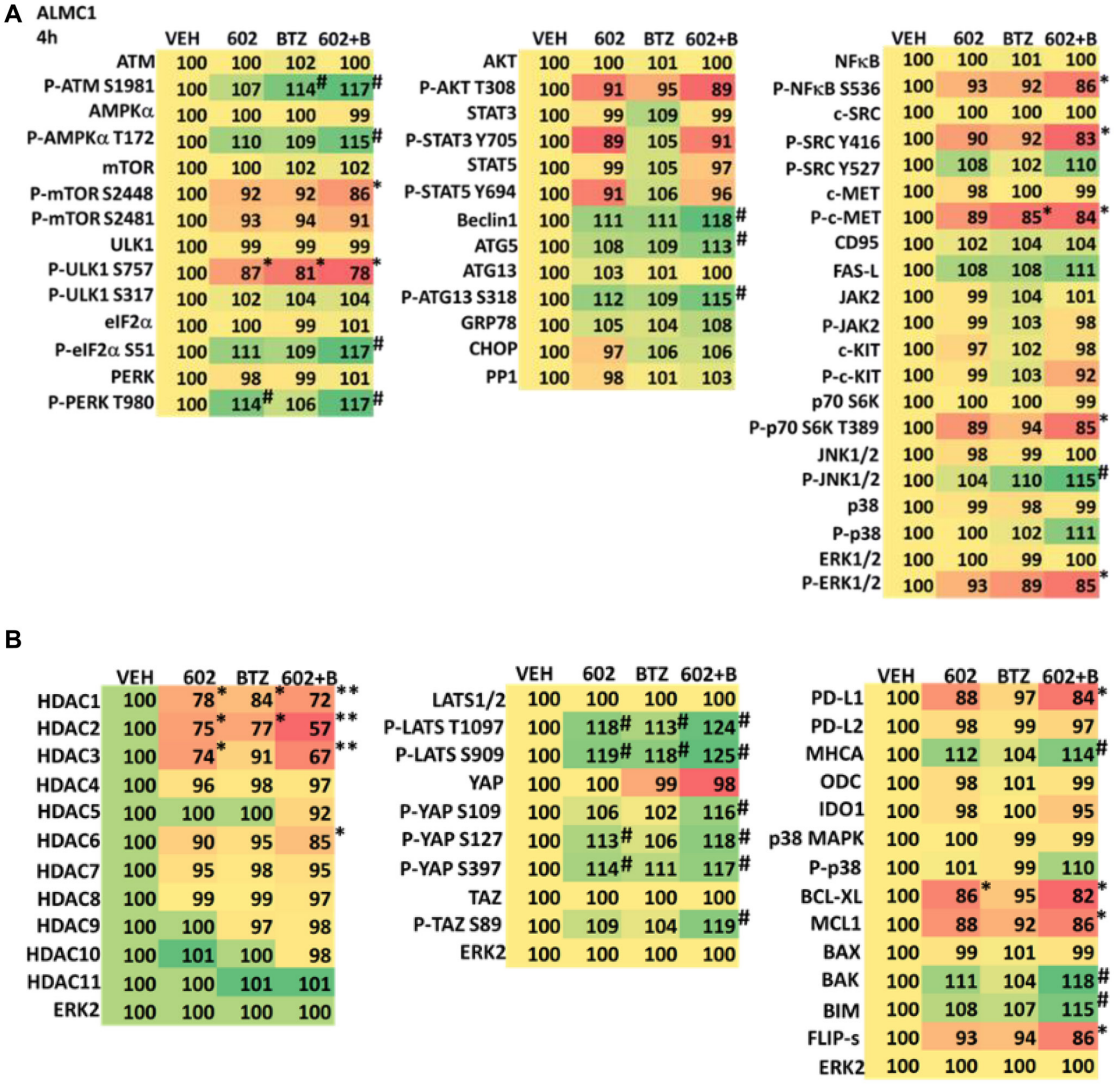
(**A**) GZ17-6.02 and bortezomib regulate signaling pathways in ALMC1 cells. Cells were treated with vehicle control, GZ17-6.02 (2 μM, curcumin final), bortezomib (10 nM) or the drugs in combination for 4 h. Cells were centrifuged and fixed *in situ*, permeabilized, stained with the indicated validated primary antibodies and imaged with secondary antibodies carrying red- and green-fluorescent tags. The staining intensity of at least 100 cells per well/condition is determined in three separate studies. The data are the normalized amount of fluorescence set at 100% comparing intensity values for vehicle control (*n* = 3 +/− SD). ^#^ *p* < 0.05 greater than vehicle control; ^*^ *p* < 0.05 less than vehicle control. Tabular data are presented as a heat-map (Microsoft Excel: conditional formatting, color scales: green, yellow, red). Green indicates greater levels and red indicates lower levels of staining. (**B**) GZ17-6.02 and bortezomib regulate signaling pathways in ALMC1 cells. Cells were treated with vehicle control, GZ17-6.02 (2 μM, curcumin final), bortezomib (10 nM) or the drugs in combination for 4 h. Cells were centrifuged and fixed *in situ*, permeabilized, stained with the indicated validated primary antibodies and imaged with secondary antibodies carrying red- and green-fluorescent tags. The staining intensity of at least 100 cells per well/condition is determined in three separate studies. The data are the normalized amount of fluorescence set at 100% comparing intensity values for vehicle control (*n* = 3 +/− SD). ^#^ *p* < 0.05 greater than vehicle control; ^*^ *p* < 0.05 less than vehicle control. Tabular data are presented as a heat-map (Microsoft Excel: conditional formatting, color scales: green, yellow, red). Green indicates greater levels and red indicates lower levels of staining.

**Figure 5:**
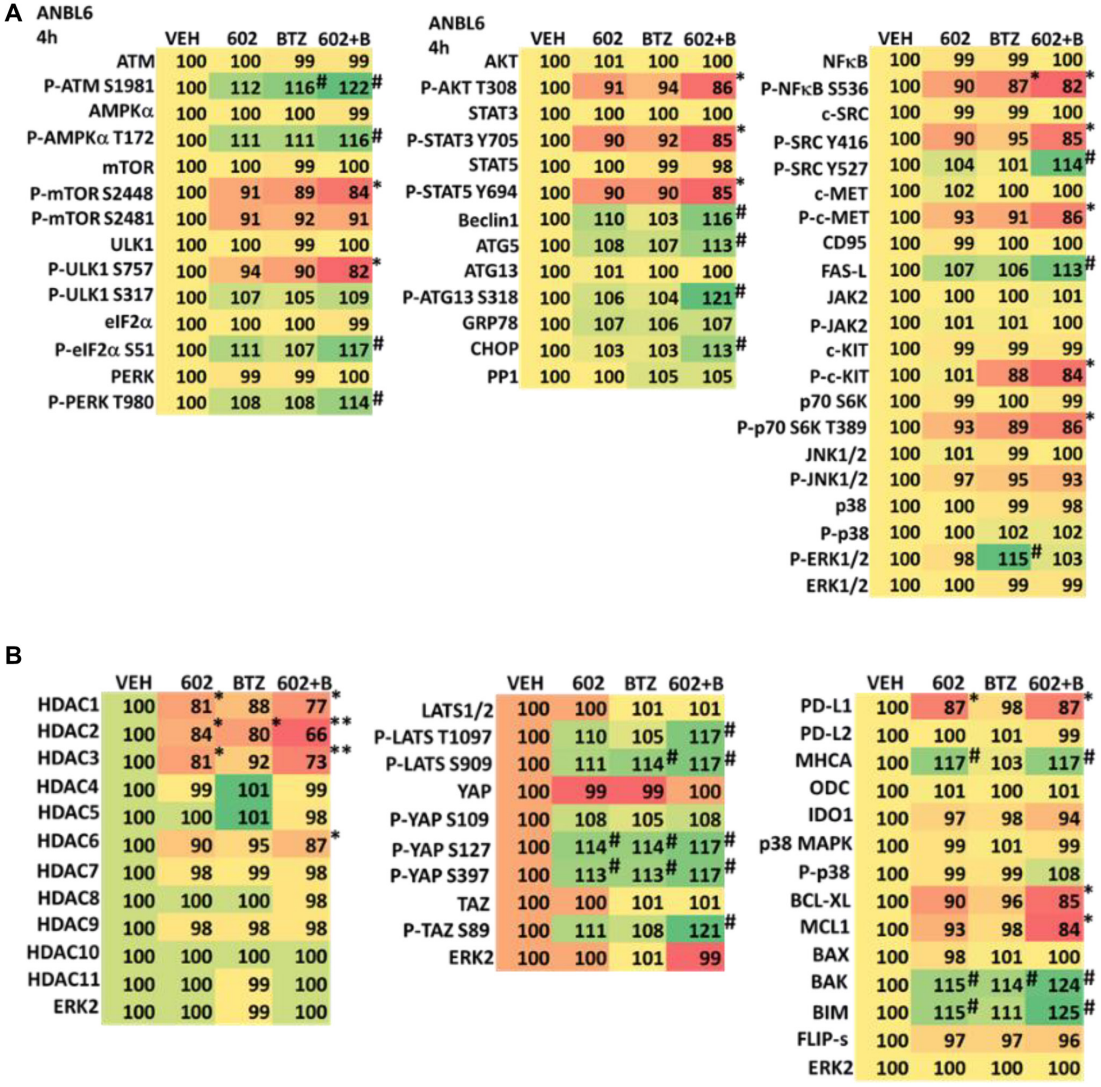
(**A**) GZ17-6.02 and bortezomib regulate signaling pathways in ANBL6 cells. Cells were treated with vehicle control, GZ17-6.02 (2 μM, curcumin final), bortezomib (10 nM) or the drugs in combination for 4 h. Cells were centrifuged and fixed *in situ*, permeabilized, stained with the indicated validated primary antibodies and imaged with secondary antibodies carrying red- and green-fluorescent tags. The staining intensity of at least 100 cells per well/condition is determined in three separate studies. The data are the normalized amount of fluorescence set at 100% comparing intensity values for vehicle control (*n* = 3 +/− SD). ^#^ *p* < 0.05 greater than vehicle control; ^*^ *p* < 0.05 less than vehicle control. Tabular data are presented as a heat-map (Microsoft Excel: conditional formatting, color scales: green, yellow, red). Green indicates greater levels and red indicates lower levels of staining. (**B**) GZ17-6.02 and bortezomib regulate signaling pathways in ANBL6 cells. Cells were treated with vehicle control, GZ17-6.02 (2 μM, curcumin final), bortezomib (10 nM) or the drugs in combination for 4 h. Cells were centrifuged and fixed *in situ*, permeabilized, stained with the indicated validated primary antibodies and imaged with secondary antibodies carrying red- and green-fluorescent tags. The staining intensity of at least 100 cells per well/condition is determined in three separate studies. The data are the normalized amount of fluorescence set at 100% comparing intensity values for vehicle control (*n* = 3 +/− SD). ^#^ *p* < 0.05 greater than vehicle control; ^*^ *p* < 0.05 less than vehicle control. Tabular data are presented as a heat-map (Microsoft Excel: conditional formatting, color scales: green, yellow, red). Green indicates greater levels and red indicates lower levels of staining.

**Figure 6:**
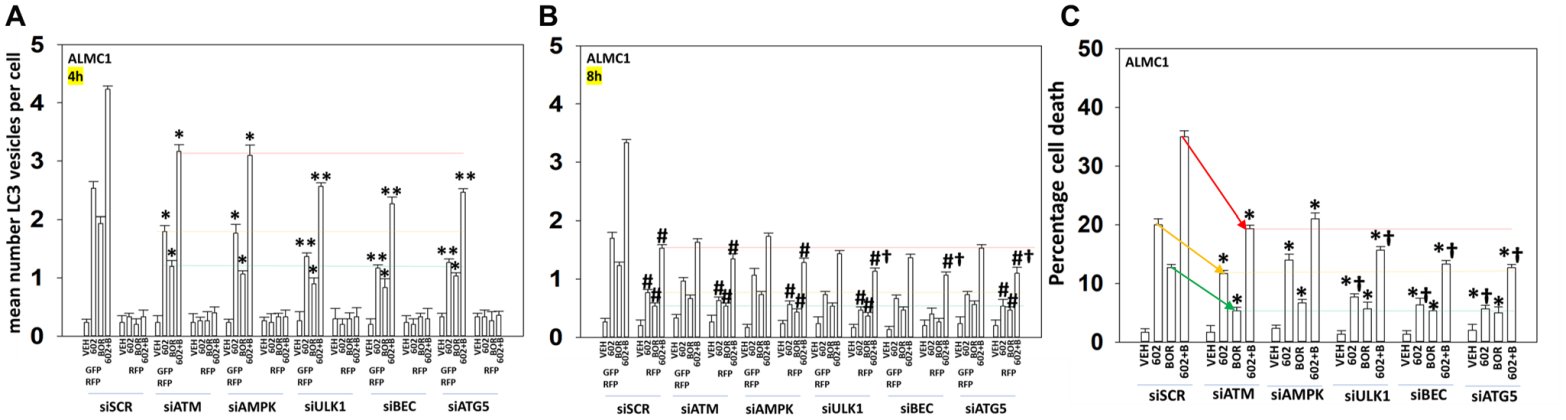
Signaling by ATM-AMPK and autophagosome formation are toxic events when cells are treated with GZ17-6.02 and bortezomib. (**A**, **B**) ALMC1 cells were transfected with a plasmid to express LC3-GFP-RFP and co-transfected with a scrambled siRNA or with siRNA molecules to knock down the expression of ATM, AMPKα, ULK1, Beclin1 or ATG5. Twenty-four h later, cells were treated with vehicle control, GZ17-6.02 (curcumin 2 μM, final), bortezomib (10 nM) or the drugs in combination for 4 h and 8 h. At each time point, the mean number of autophagosomes (GFP+ RFP+) and autolysosomes (RFP+) per cell were determined randomly in >100 cells. (*n* = 3 +/− SD). ^*^ *p* < 0.05 less than corresponding value at 4 h; ^**^ *p* < 0.05 less than corresponding values in siATM and siAMPK transfected cells; ^#^ *p* < 0.05 greater than corresponding value at 4 h; ^†^ *p* < 0.05 less than corresponding values in siATM and siAMPK transfected cells. (**C**) ALMC1 cells were transfected with a scrambled siRNA or with siRNA molecules to knock down the expression of ATM, AMPKα, ULK1, Beclin1 or ATG5. Twenty-four h later, cells were treated with vehicle control, GZ17-6.02 (curcumin 2 μM, final), bortezomib (10 nM) or the drugs in combination for 24 h. Twenty-four h later, cells were isolated, and viability determined via trypan blue exclusion assays (*n* = 3 +/− SD). ^*^ *p* < 0.05 less than corresponding value in siSCR cells; ^†^ *p* < 0.05 less than corresponding values in siATM and siAMPK transfected cells.

**Figure 7:**
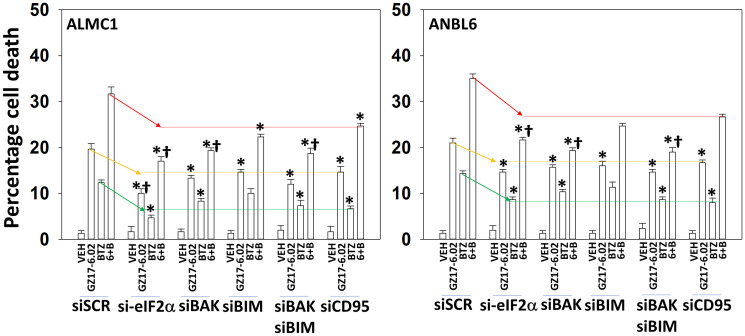
ER stress signaling plays an important role in mediating GZ17-6.02/bortezomib lethality. ALMC1 and ANBL6 cells were transfected with scrambled siRNA or with siRNA molecules to knock down eIF2α, BAK, BIM or CD95. After 24 h, cells were treated with vehicle control, GZ17-6.02 (2 μM curcumin, final), bortezomib (10 nM) or the drugs in combination for 24 h. Twenty-four h later, cells were isolated, and viability determined via trypan blue exclusion assays (*n* = 3 +/− SD). ^*^ *p* < 0.05 less than corresponding value in siSCR cells; ^†^ *p* < 0.05 less than corresponding values in siCD95 transfected cells.

We determined the impact GZ17-6.02 had on cell signaling processes in the MM cells. The drug combination of GZ17-6.02 and bortezomib in MM cells activated ATM, the AMPK and PERK and inactivated ULK1, mTORC1, eIF2α, NFκB and the Hippo pathway (Figures 4A, 4B and 5A, 5B; Supplementary Figure 3A, 3B). The drug combination increased ATG13 S318 phosphorylation and the expression of Beclin1, ATG5, BAK and BIM, and reduced the levels of BCL-XL and MCL1. Collectively, these alterations would predict for enhance tumor cell death. GZ17-6.02 interacted with bortezomib to enhance autophagosome formation and autophagic flux, and knock down of ATM, AMPKα, ULK1, Beclin1 or ATG5 significantly reduced both autophagy and tumor cell killing (Figure 6 and Supplementary Figure 4). This data, with respect to the findings in Supplementary Figure 2, also suggests that in multiple myeloma cells autophagosome formation caused by GZ17-6.02 initiates a pathway towards cell death that does not per se involve the formation of autolysosomes and autophagic flux. Knock down of eIF2α, BAK, BIM or CD95 significantly reduced tumor cell killing (Figure 7). Knock down of eIF2α or BAK exhibited the greatest cytoprotective effects against GZ17-6.02 as a single agent and when combined with bortezomib when compared to the knock down of other proteins.

**Figure 8:**
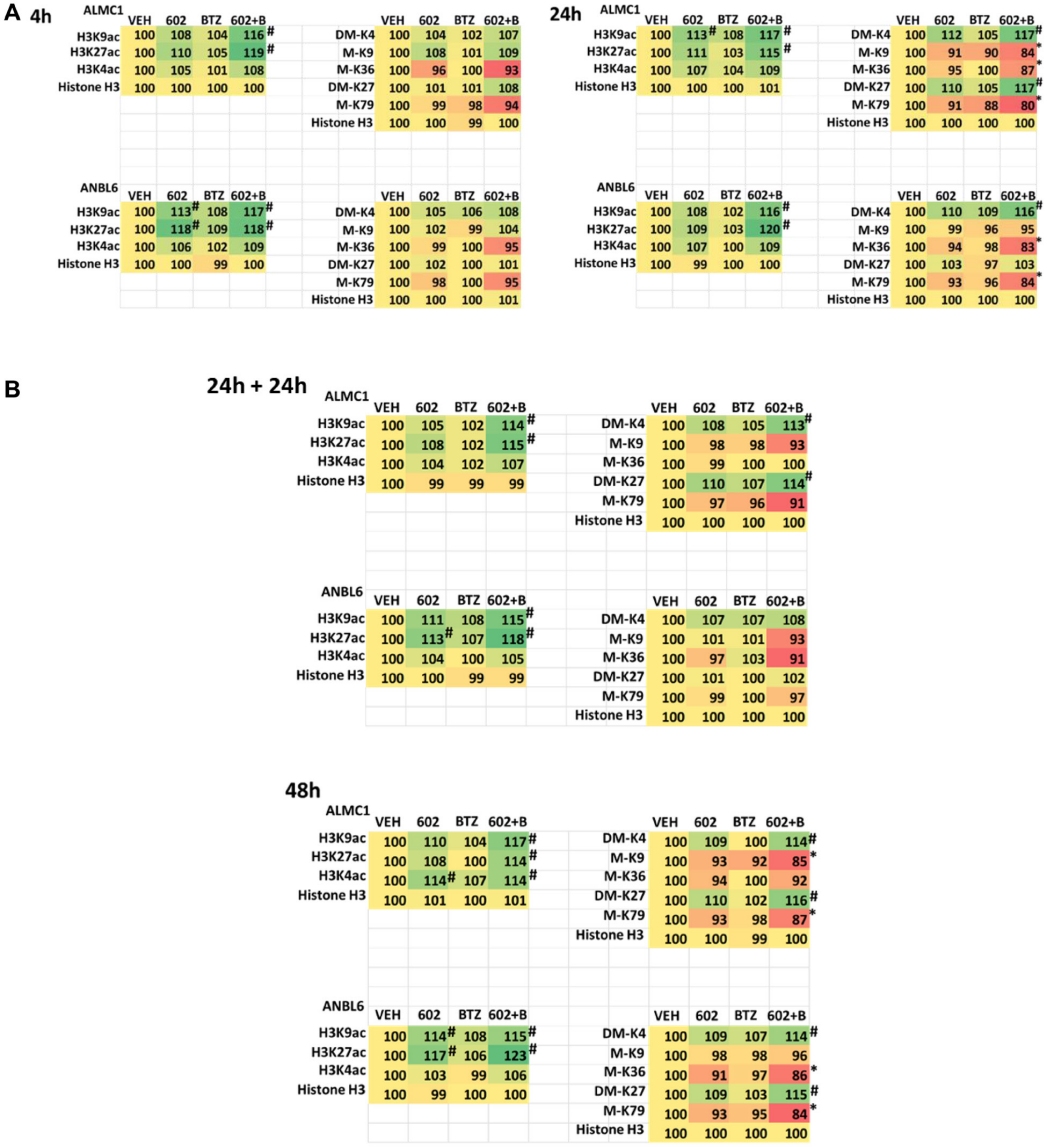
(**A**) GZ17-6.02 and bortezomib regulate histone H3 acetylation and methylation in multiple myeloma cells. ALMC1 and ANBL6 cells were treated with vehicle control, GZ17-6.02 (2 μM, curcumin final), bortezomib (10 nM) or the drugs in combination for 4 h. Cells were centrifuged and fixed *in situ*, permeabilized, stained with the indicated validated primary antibodies and imaged with secondary antibodies carrying red- and green-fluorescent tags. The staining intensity of at least 100 cells per well/condition is determined in three separate studies. The data are the normalized amount of fluorescence set at 100% comparing intensity values for vehicle control (*n* = 3 +/− SD). ^#^ *p* < 0.05 greater than vehicle control; ^*^ *p* < 0.05 less than vehicle control. Tabular data are presented as a heat-map (Microsoft Excel: conditional formatting, color scales: green, yellow, red). Green indicates greater levels and red indicates lower levels of staining. (**B**) GZ17-6.02 and bortezomib regulate histone H3 acetylation and methylation in multiple myeloma cells. ALMC1 and ANBL6 cells were treated with vehicle control, GZ17-6.02 (2 μM, curcumin final), bortezomib (10 nM) or the drugs in combination for 4 h. Cells were centrifuged and fixed *in situ*, permeabilized, stained with the indicated validated primary antibodies and imaged with secondary antibodies carrying red- and green-fluorescent tags. The staining intensity of at least 100 cells per well/condition is determined in three separate studies. The data are the normalized amount of fluorescence set at 100% comparing intensity values for vehicle control (*n* = 3 +/− SD). ^#^ *p* < 0.05 greater than vehicle control; ^*^ *p* < 0.05 less than vehicle control. Tabular data are presented as a heat-map (Microsoft Excel: conditional formatting, color scales: green, yellow, red). Green indicates greater levels and red indicates lower levels of staining.

The expression of HDACs1/2/3 was significantly reduced by GZ17-6.02 beyond that previously observed in prostate cancer cells and, this observation required macroautophagy (Figures 4A, 4B and 5A, 5B; Supplementary Figures 5 and 6) [15–23]. This was associated with changes in the acetylation, methylation, and phosphorylation of histone H3 at multiple NH_2_-terminal regulatory sites (Figure 8A, 8B and Supplementary Figure 7). The acetylation of histone H3 at K9 and K27 was enhanced by the drug combination after 4 h, and this was maintained for 48 h, even in cells that had spent 24 h in the absence of drugs. Changes in histone H3 methylation were observed after 24 h and 48 h, with methylation levels both increasing and decreasing. After 24 h, in both lines, the di-methylation of histone H3 K4 was enhanced and the mono-methylation of K36 and K79 declined. After 48 h, the di-methylation of K4 remained elevated and the di-methylation of K27 was also increased. The methylation of K79 remained lower.

GZ17-6.02 also increased the phosphorylation of histone H3. The phosphorylation of histone H3 T3 observed after 4 h and was maintained over the 48 h time course. The phosphorylation of histone H3 S10 was elevated after 4 h, but then declined in ALMC1 cells whereas it was maintained in ANBL6 cells. The phosphorylation of histone H3 S28 was transiently enhanced after 24 h only in ALMC1 cells. The phosphorylation of histone H3 T11 was unaltered at any time point in either line.

**Figure 9:**
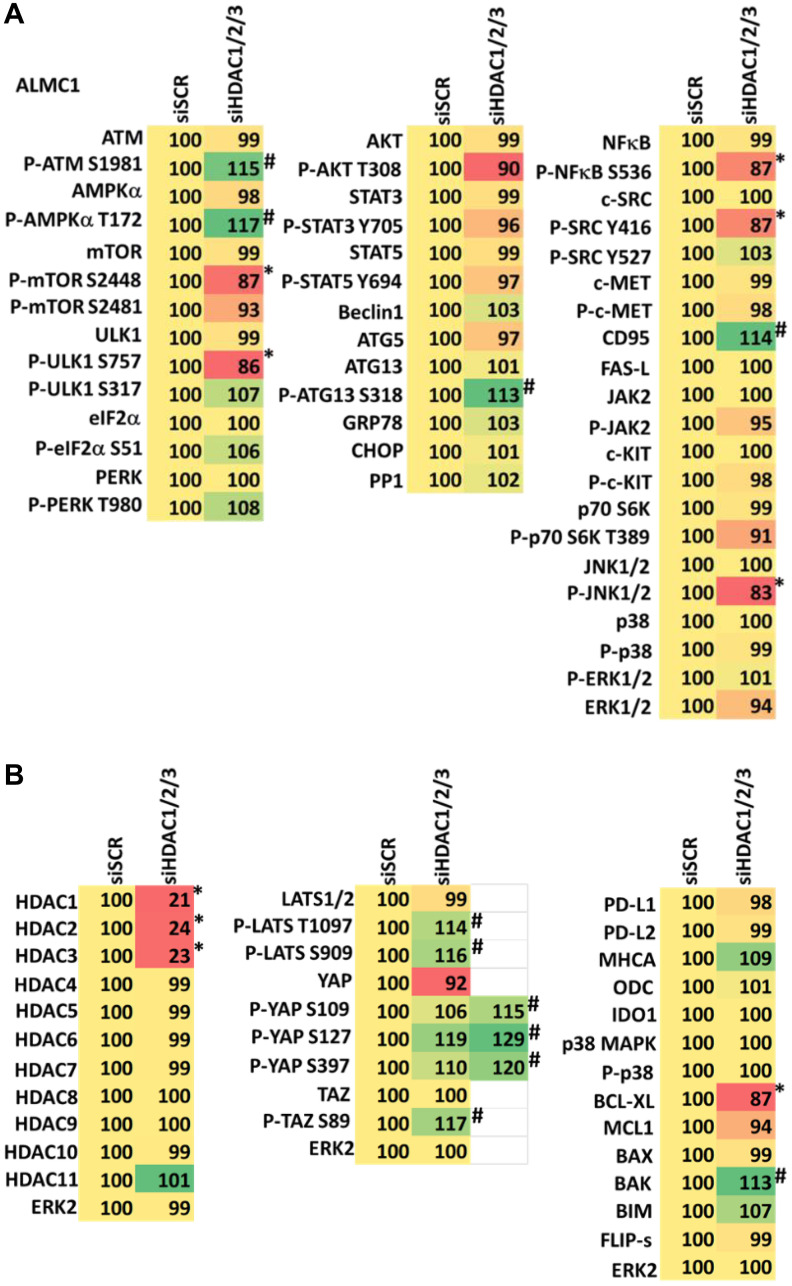
(**A**) Knock down of HDACs1/2/3 regulates signaling in ALMC1 cells in a fashion similar to that of GZ17-6.02. Cells were transfected with a scrambled siRNA or with siRNAs combined to knock down the expression of HDACs1/2/3. After 12 h cells were centrifuged and fixed *in situ*, permeabilized, stained with the indicated validated primary antibodies and imaged with secondary antibodies carrying red- and green-fluorescent tags. The staining intensity of at least 100 cells per well/condition is determined in three separate studies. The data are the normalized amount of fluorescence set at 100% comparing intensity values for vehicle control (*n* = 3 +/− SD). ^#^ *p* < 0.05 greater than vehicle control; ^*^ *p* < 0.05 less than vehicle control. Tabular data are presented as a heat-map (Microsoft Excel: conditional formatting, color scales: green, yellow, red). Green indicates greater levels and red indicates lower levels of staining. (**B**) Knock down of HDACs1/2/3 regulates signaling in ALMC1 cells in a fashion similar to that of GZ17-6.02. Cells were transfected with a scrambled siRNA or with siRNAs combined to knock down the expression of HDACs1/2/3. After 12 h cells were centrifuged and fixed *in situ*, permeabilized, stained with the indicated validated primary antibodies and imaged with secondary antibodies carrying red- and green-fluorescent tags. The staining intensity of at least 100 cells per well/condition is determined in three separate studies. The data are the normalized amount of fluorescence set at 100% comparing intensity values for vehicle control (*n* = 3 +/− SD). ^#^ *p* < 0.05 greater than vehicle control; ^*^ *p* < 0.05 less than vehicle control. Tabular data are presented as a heat-map (Microsoft Excel: conditional formatting, color scales: green, yellow, red). Green indicates greater levels and red indicates lower levels of staining.

**Figure 10:**
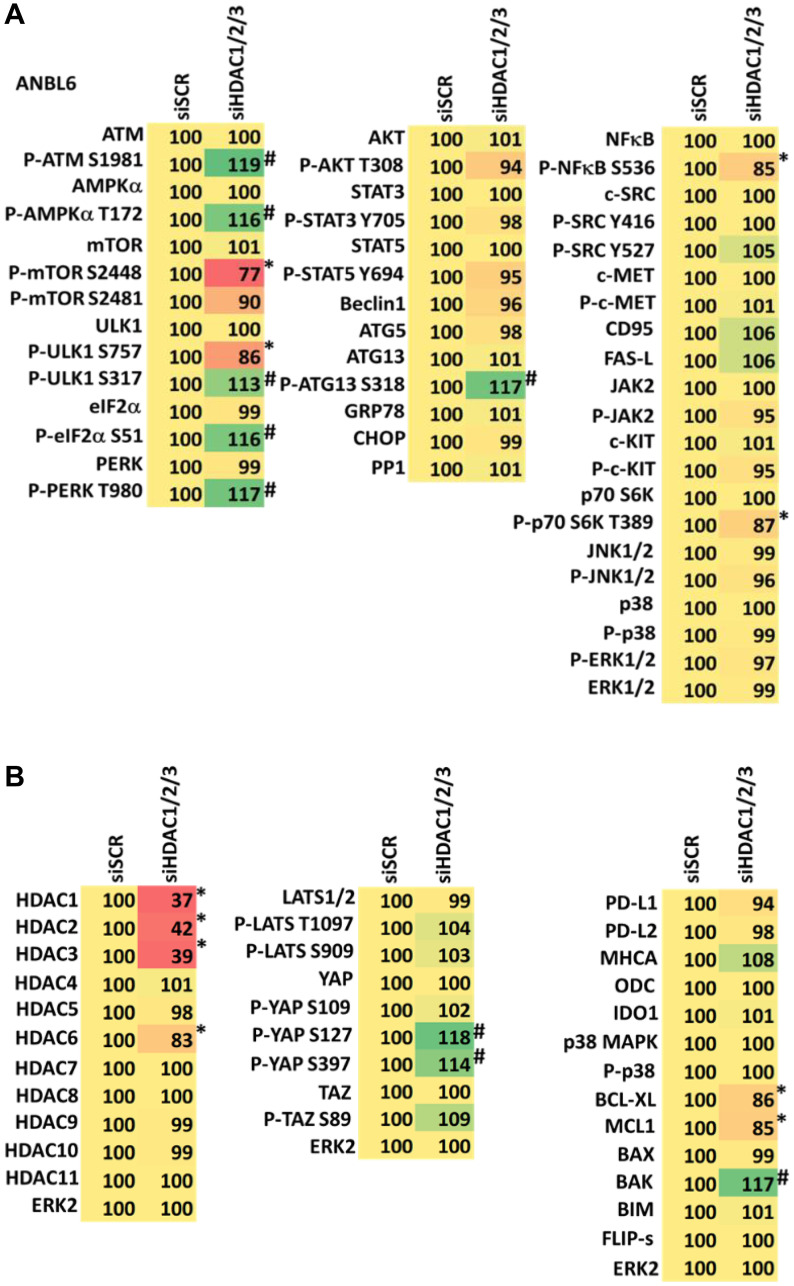
(**A**) Knock down of HDACs1/2/3 regulates signaling in ANBL6 cells in a fashion similar to that of GZ17-6.02. Cells were transfected with a scrambled siRNA or with siRNAs combined to knock down the expression of HDACs1/2/3. After 12 h cells were centrifuged and fixed *in situ*, permeabilized, stained with the indicated validated primary antibodies and imaged with secondary antibodies carrying red- and green-fluorescent tags. The staining intensity of at least 100 cells per well/condition is determined in three separate studies. The data are the normalized amount of fluorescence set at 100% comparing intensity values for vehicle control (*n* = 3 +/− SD). ^#^ *p* < 0.05 greater than vehicle control; ^*^ *p* < 0.05 less than vehicle control. Tabular data are presented as a heat-map (Microsoft Excel: conditional formatting, color scales: green, yellow, red). Green indicates greater levels and red indicates lower levels of staining. (**B**) Knock down of HDACs1/2/3 regulates signaling in ANBL6 cells in a fashion similar to that of GZ17-6.02. Cells were transfected with a scrambled siRNA or with siRNAs combined to knock down the expression of HDACs1/2/3. After 12 h cells were centrifuged and fixed *in situ*, permeabilized, stained with the indicated validated primary antibodies and imaged with secondary antibodies carrying red- and green-fluorescent tags. The staining intensity of at least 100 cells per well/condition is determined in three separate studies. The data are the normalized amount of fluorescence set at 100% comparing intensity values for vehicle control (*n* = 3 +/− SD). ^#^ *p* < 0.05 greater than vehicle control; ^*^ *p* < 0.05 less than vehicle control. Tabular data are presented as a heat-map (Microsoft Excel: conditional formatting, color scales: green, yellow, red). Green indicates greater levels and red indicates lower levels of staining.

Combined knock down of HDACs1/2/3 caused activation of ATM and the AMPK and caused inactivation of ULK1, mTORC1, NFκB and the Hippo pathway (Figures 9A, 9B and 10A, 10B). HDAC knock down also enhanced ATG13 S318 phosphorylation, i.e., autophagosome formation, increased BAK levels and reduced those of BCL-XL, although it did not enhance endoplasmic reticulum stress signaling as judged by eIF2α S51 phosphorylation. Thus, GZ17-6.02 initially causes a series of signaling events which leads to autophagic degradation of HDACs 1, 2 and 3, which then directly induces, as a secondary event, many of the same alterations in cell signaling within the multiple myeloma cells caused by GZ17-6.02, ultimately causing tumor cell death.

## DISCUSSION

We recently demonstrated that GZ17-6.02 was highly efficacious at suppressing the growth of LNCaP prostate tumors in mice, significantly prolonging survival weeks beyond the cessation of drug exposure [21]. The present studies were performed to extend our knowledge of GZ17-6.02 biology from that known in solid tumor cell types such as prostate cancer cells to liquid tumor cell types, for example, multiple myeloma. Many of the biological processes engaged by GZ17-6.02 in solid tumor cells were also found to operate in MM cells, however, there were also notable differences. For example, the ability of GZ17-6.02 to reduce the expression of HDACs1/2/3 was enhanced in the MM cells compared to solid tumor cells whereas the degradation of HDAC6 was reduced. GZ17-6.02 promoted greater levels of autophagosome formation in MM cells compared to solid tumor cell types, but the amount of autophagic flux to autolysosomes was not significantly enhanced. GZ17-6.02 was significantly better at killing MM cells than prostate cancer cells, and in both tumor types, knock down of ULK1, Beclin1 or ATG5 reduced both autophagosome formation and tumor cell killing.

*Multiple myeloma cells made resistant to bortezomib remained sensitive to being killed by GZ17-6.02 with no significant difference comparing parental and resistant cells.* The reasons for this observation are presently unclear, in part, because the studies in our initial report have been focused on developing an initial understanding of GZ17-6.02 biology in parental cells and its interaction with bortezomib. Several publications have argued that bortezomib-induced autophagy acts to reduce drug efficacy [30–34]. Other groups, however, state that drug-induced autophagy results in MM cell apoptosis [35, 36]. Our data demonstrated that GZ17-6.02 and bortezomib had similar overlapping mechanisms regulating cell killing, supporting the notion that autophagy feeds into apoptosis. Bortezomib-induced cell death was significantly reduced by knock down of ATM, AMPKα, ULK1, Beclin1 or ATG5, with all knock downs reducing the amount of killing to the same extent. In contrast for GZ17-6.02 as a single agent or when combined with bortezomib, a slightly different biology was observed. Knock down of ATM or AMPKα significantly reduced killing whereas knock down of ULK1, Beclin1 or ATG5 caused a further additional significant reduction in cell death. Unlike solid tumor cells where GZ17-6.02 as a single agent always was observed to cause significant amounts of ATM activation, this effect was not observed in MM cells, and unexpectedly, we found that bortezomib as a single agent activated ATM. Studies beyond the scope of this manuscript will be required to study bortezomib-induced ATM activation in parental cells and in bortezomib-resistant cells.

Based on the large reductions in HDAC1/2/3 expression caused by GZ17-6.02 as a single agent and more so when combined with bortezomib, we determined whether the acetylation and methylation of lysine residues within histone H3 were altered. The acetylation of histone H3 at lysine 9 and at lysine 27 was enhanced within 4 h by GZ17-6.02 as a single agent and this persisted for 48 h; even in cells cultured in the absence of the drug for 24 h, acetylation at these sites was maintained. In contrast, no changes in histone H3 methylation were observed after 4 h of treatment, and after 24 h, in both cell lines tested, the di-methylation of lysine 4 was elevated only by the drug combination and the methylation of lysine 36 and lysine 79 reduced, again only by the drug combination. After 48 h, in both lines, the di-methylation of lysine 4 remained high as was di-methylation of lysine 27. The methylation of lysine 79 in both lines remained lower.

Acetylation and methylation of histone H3 has been linked in multiple tumor cell types to the regulation of transcription, and GZ17-6.02 has been linked to the regulation of super-enhancer elements [37]. Mithraprabhu et al. and Yuan et al. both demonstrated that MM cells are sensitive in the laboratory and in patients to histone deacetylase inhibitors [38, 39]. Elevated histone H3 lysine 27 acetylation is indicative of gene activation in multiple myeloma cells and was associated with poorer patient survival [40]. Silencing of the transcription factor ELK1 was associated with decreased histone H3 lysine 9 acetylation, increased lysine 9 methylation and reduced expression of the pluripotency gene Oct4 [41]. Changes in histone methylation were delayed secondary events compared to alterations in acetylation. Di- and tri-methylation of lysine 4 are markers of transcriptional activation and we observed prolonged di-methylation of lysine 4 after 24 h of GZ17-6.02 and bortezomib exposure [42]. Reduced lysine 79 methylation would be predicted to correlate with reduced transcription [43, 44]. Increased K79 methylation has also been linked to UV-induced recombination repair and cell cycle checkpoint activation, which potentially links our observation that knock down of HDAC proteins activated ATM [45].

GZ17-6.02 as a single agent and when combined with bortezomib enhanced phosphorylation of threonine 3 in both lines and this was maintained for 48 h. Phosphorylation of T3 has been linked to chromosome segregation, chromosome methylation and signaling by the Aroura B kinase/protein phosphatase 1 complex [46–49]. For example, the demethylase Dnmt3a interacts with the H3 histone tail and binding can be disrupted by di- and trimethylation of K4, acetylation of K4 and by phosphorylation of T3. In addition to these findings is that Aurora B is the catalytic ‘kinase’ subunit which coordinates mitosis. In prometaphase, the Aroura B kinase/protein phosphatase 1/Haspin complex is enriched at centromeres and controls spindle checkpoint and kinetochore-microtubule interactions. Future studies will need to address the relevance of the alterations in acetylation, methylation, and phosphorylation to the MM biology of GZ17-6.02.

Knock down of eIF2α significantly reduced the lethality of GZ17-6.02 and of bortezomib as single agents, and more so when cells were treated with the drug combination. This correlates with our findings from both MM lines examining the phosphorylation of PERK and eIF2α where in general, it was the drug combination that was required to generate a significant endoplasmic reticulum response [15–23]. In MM the PERK-eIF2α-ATF4-CHOP ER stress response has been linked by many groups to elevated tumor cell killing [50–52]. As noted earlier, many groups have also linked macroautophagy to proteasome inhibitor resistance, including autophagy stimulated by ER stress [53]. Our data demonstrated that autophagosome formation and MM cell killing by the drug combination was most effectively reduced by knocking down the expression of ULK1, Beclin1 or ATG5, strongly arguing that macroautophagy played a key role in tumor cell execution. As a single agent and as previously observed in solid tumor cells, GZ17-6.02 enhanced macroautophagy which was essential for tumor cell killing. However, we did not expect cell killing by bortezomib as a single agent to also be reduced by knock down of ULK1, Beclin1 or ATG5. Reasons for the difference between our data and other groups may be linked to the relatively low concentrations of bortezomib used in our studies, and that our genetic manipulations were transient rather than stable knock downs.

GZ17-6.02 and bortezomib activated LATS1/2 and increased the phosphorylation of YAP and TAZ in the Hippo pathway; YAP and TAZ phosphorylation causes their nuclear exit and prevents them from acting as co-transcription factors with TEADS proteins in the nucleus [54–61]. In solid tumor cell types, nuclear-localized YAP and TAZ play key roles in promoting tumor cell growth, metastatic spread, and resistance to chemotherapy. In MM cells, the literature argues both for YAP and TAZ acting under some circumstances as tumor promoters and situationally acting as tumor suppressors. YAP can associate with both acetylases and methylases to alter chromatin structure and transcription. Studies beyond the present manuscript will be required to understand the relevance of the Hippo pathway to the biology of GZ17-6.02 in MM cells.

## MATERIALS AND METHODS

### Materials

The ALMC1, ANBL6 and U266 multiple myeloma cell lines were purchased from the ATCC (Bethesda, MD, USA). Bortezomib and carfilzomib were purchased from Selleckchem (Houston, TX, USA). All Materials were obtained as described in the references [14–23]. Trypsin-EDTA, DMEM, RPMI, penicillin-streptomycin were purchased from GIBCOBRL (GIBCOBRL Life Technologies, Grand Island, NY, USA). Other reagents and performance of experimental procedures were as described [14–23]. Antibodies were purchased from Cell Signaling Technology (Danvers, MA, USA); Abgent (San Diego, CA, USA); Novus Biologicals (Centennial, CO, USA); Abcam (Cambridge, UK); and Santa Cruz Biotechnology (Dallas, TX, USA). Specific multiple independent siRNAs to knock down the expression of CD95, Beclin1, ATG5, AMPKα_1_, ATM, BIM, BAX, BAK, BID and eIF2α, and scramble control, were purchased from Qiagen (Hilden, Germany) and Thermo Fisher (Waltham, MA, USA). Control studies were presented in prior manuscripts showing on-target specificity of our siRNAs, primary antibodies, and our phospho-specific antibodies to detect both total protein levels and phosphorylated levels of proteins [14–23] (Supplementary Figure 8).

### Methods

All bench-side Methods used in this manuscript have been previously performed and described in the peer-reviewed references [14–23].

### Assessments of protein expression and protein phosphorylation [14–23]

At various time-points after the initiation of drug exposure, cells in 96-well plates are fixed in place using paraformaldehyde and using Triton X100 for permeabilization. Standard immunofluorescent blocking procedures are employed, followed by incubation of different wells with a variety of validated primary antibodies and subsequently validated fluorescent-tagged secondary antibodies are added to each well. Assessments of staining intensity were made using a Hermes wide field microscope (Idea Biotechnology, Rehovot, Israel) using its internal software. Tabular data are presented as a heat-map (Microsoft Excel: conditional formatting, color scales: green, yellow, red). Green indicates greater levels and red indicates lower levels of staining.

### Detection of cell death by trypan blue assay [14–23]

Cells were treated with vehicle control or with drugs alone or in combination for 24 h. At the indicated time points cells were harvested by trypsinization and centrifugation. Cell pellets were resuspended in PBS and mixed with trypan blue agent. Viability was determined microscopically using a hemocytometer. Five hundred cells randomly chosen from the four fields of the hemocytometer were counted and the number of dead cells was counted and expressed as a percentage of the total number of cells counted.

### Transfection of cells with siRNA [14–23]

Cells were plated and 24 h after plating, transfected. A plasmid to express LC3-GFP-RFP was used throughout the study (Addgene, Waltham, MA). For siRNA transfection, 10 nM of the annealed siRNA or the negative control (a “scrambled” sequence with no significant homology to any known gene sequences from mouse, rat, or human cell lines) were used.

### Assessments of autophagosome and autolysosome levels [14–23]

Cells were transfected with a plasmid to express LC3-GFP-RFP (Addgene, Watertown MA). Twenty-four hours after transfection, cells are treated with vehicle control or the drugs alone or in combination. Cells were imaged and recorded at 60X magnification 4 h and 8 h after drug exposure and the mean number of (GFP+RFP+) and (RFP+) punctae per cell determined from >100 randomly selected cells per condition.

### Data analysis

Comparison of the effects of various treatments was using one-way ANOVA for normalcy followed by a two tailed Student’s *t*-test with multiple comparisons. Differences with a *p*-value of < 0.05 were considered statistically significant. Experiments are the means of multiple individual data points per experiment from 3 independent experiments (± SD).

## SUPPLEMENTARY MATERIALS



## Abbreviations

ERK: extracellular regulated kinase
PI3K: phosphatidyl inositol 3 kinase
ca: constitutively active
dn: dominant negative
ER: endoplasmic reticulum
AMPK: AMP-dependent protein kinase
mTOR: mammalian target of rapamycin
JAK: Janus Kinase
STAT: Signal Transducers and Activators of Transcription
MAPK: mitogen activated protein kinase
PTEN: phosphatase and tensin homologue on chromosome ten
ROS: reactive oxygen species
CMV: empty vector plasmid
si: small interfering
SCR: scrambled
VEH: vehicle
602: GZ17-6.02
BTZ: bortezomib
MHCA: major histocompatibility class A
