# Supplementary material for: GZ17-6.02 interacts with proteasome inhibitors to kill multiple myeloma cells
Source: Oncotarget. 2024 Mar 5;15:159–74. doi: 10.18632/oncotarget.28558 (PMC10913917; doi:10.18632/oncotarget.28558)
Supplement: Supplementary file 1 [file oncotarget-15-28558-s001.pdf]

GZ17-6.02 interacts with proteosome inhibitors to kill multiple myeloma cells

SUPPLEMENTARY MATERIALS

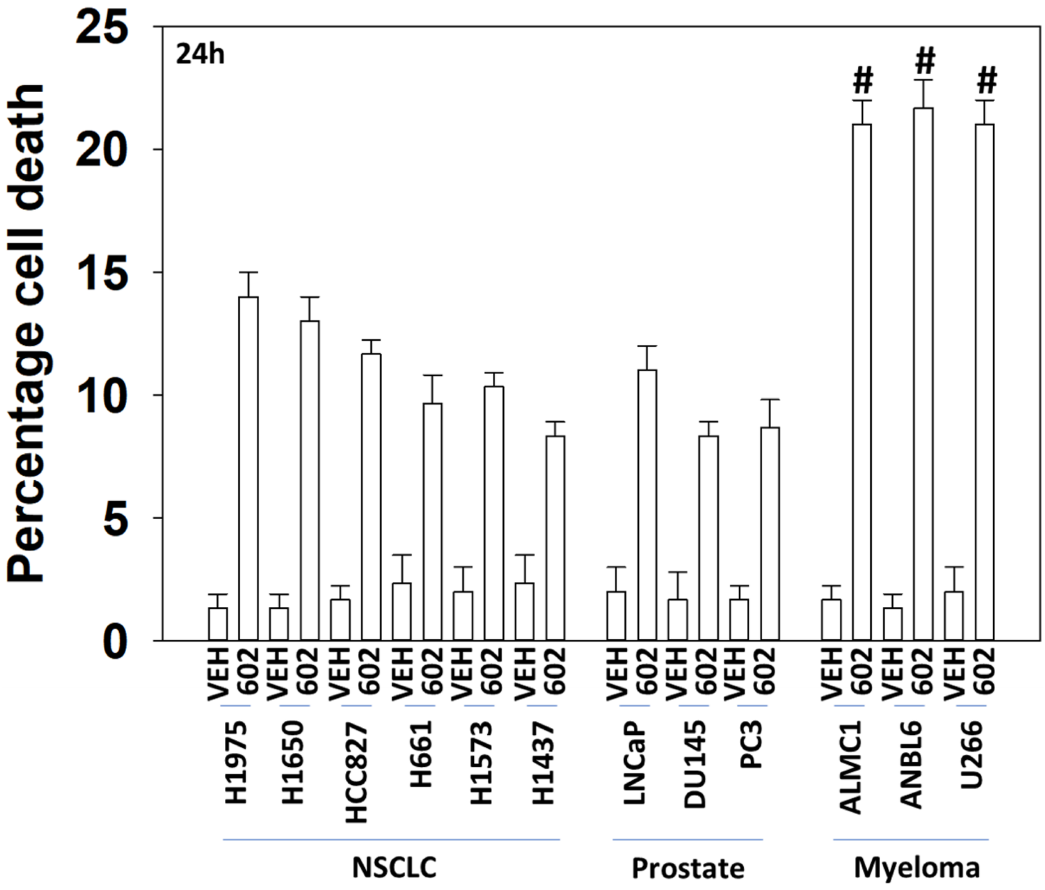

Supplementary Figure 1: Multiple myeloma cells are more effectively killed by GZ17-6.02 than prostate or lung cancer cells. Myeloma, prostate, and NSCLC cells were treated with vehicle control or with GZ17-6.02 (2 µM curcumin, final) for 24 h. Cells were isolated after 24 h and viability determined by trypan blue exclusion assay ($n = 3$ +/− SD) $^{\#}p < 0.05$ greater than corresponding values in NSCLC and prostate cancer cells.

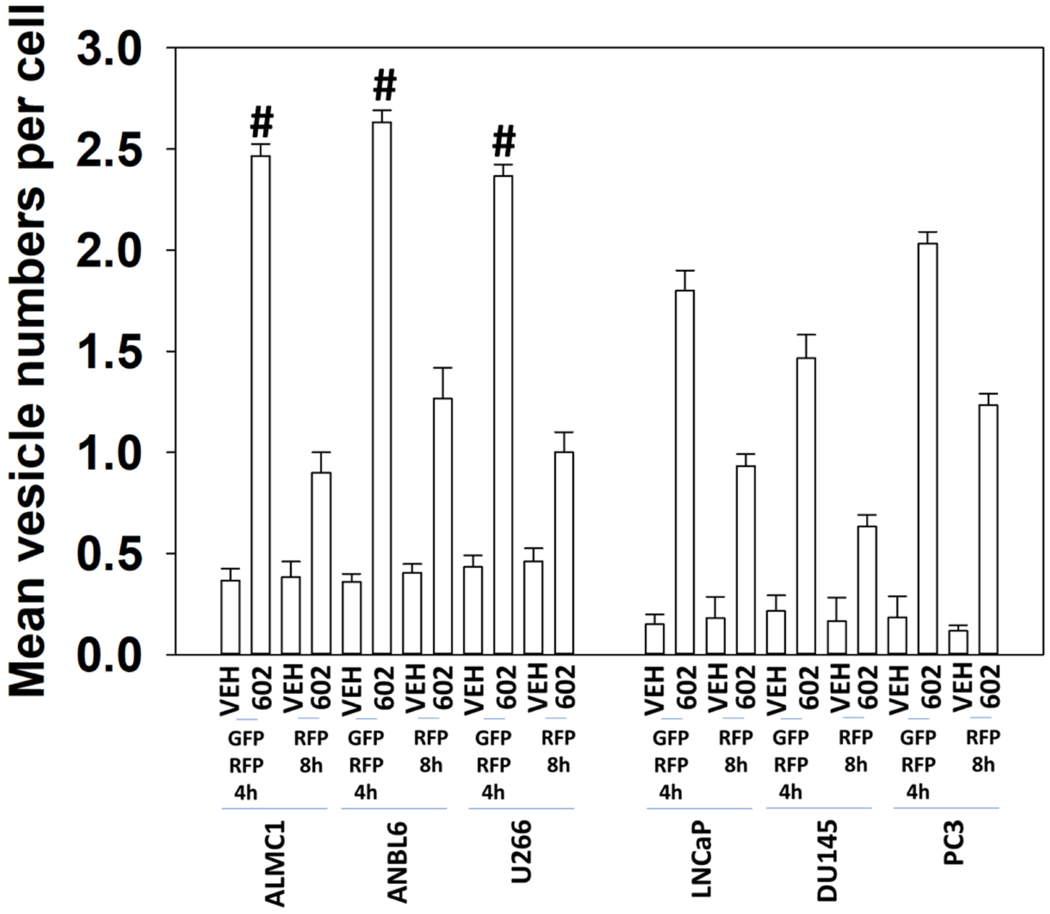

Supplementary Figure 2: GZ17-6.02 causes greater autophagosome formation in MM cells than prostate cancer cells, whereas similar levels of autolysosome formation are observed. Multiple myeloma cells and prostate cancer cells were transfected with a plasmid to express LC3-GFP-RFP. After 24 h, cells were treated with vehicle control or with GZ17-6.02 (2 μM curcumin, final) for 4 h and 8 h. At each time point, the mean number of autophagosomes (GFP+ RFP+) and autolysosomes (RFP+) per cell were determined randomly in >100 cells. (n = 3 +/− SD). $^{\#}p < 0.05$ greater than values in prostate cancer cells at 4 h.

A

U266
4h

	VEH	602	BTZ	602+B
ATM	100	100	100	100
P-ATM S1981	100	106	116#	117#
AMPKα	100	101	100	100
P-AMPKα T172	100	112	117#	121#
mTOR	100	100	101	100
P-mTOR S2448	100	86*	95	84*
P-mTOR S2481	100	87*	93	83*
ULK1	100	100	99	98
P-ULK1 S757	100	91	88	75*
P-ULK1 S317	100	108	113#	122#
eIF2α	100	101	99	100
P-eIF2α S51	100	112	117#	132#
PERK	100	99	99	100
P-PERK T980	100	111	118#	132#

	VEH	602	BTZ	602+B
AKT	100	100	100	100
P-AKT T308	100	98	100	96
STAT3	100	96	98	96
P-STAT3 Y705	100	87*	91	83*
STAT5	100	101	100	101
P-STAT5 Y694	100	95	99	94
Beclin1	100	108	103	117#
ATG5	100	107	104	112
ATG13	100	100	98	100
P-ATG13 S318	100	118#	117#	120#
GRP78	100	106	104	106
CHOP	100	98	99	102
PP1	100	100	102	105

	VEH	602	BTZ	602+B
NFκB	100	99	100	100
P-NFκB S536	100	90	87*	79*
c-SRC	100	100	97	99
P-SRC Y416	100	92	99	85*
P-SRC Y527	100	106	104	114#
c-MET	100	100	100	100
P-c-MET	100	86*	84*	82*
CD95	100	99	100	100
FAS-L	100	104	103	107
JAK2	100	99	100	100
P-JAK2	100	102	101	99
c-KIT	100	101	101	100
P-c-KIT	100	93	90	86*
p70 S6K	100	100	99	100
P-p70 S6K T389	100	97	107	95
JNK1/2	100	99	100	100
P-JNK1/2	100	97	94	94
p38	100	100	100	100
P-p38	100	100	101	102
ERK1/2	100	100	99	100
P-ERK1/2	100	95	115#	108

B

	VEH	602	BTZ	602+B
HDAC1	100	82*	91	82*
HDAC2	100	86*	86*	74*
HDAC3	100	85*	93	79*
HDAC4	100	100	101	98
HDAC5	100	99	99	99
HDAC6	100	96	99	95
HDAC7	100	99	102	100
HDAC8	100	101	100	100
HDAC9	100	99	98	99
HDAC10	100	100	100	99
HDAC11	100	101	100	100
ERK2	100	100	100	100

	VEH	602	BTZ	602+B
LATS1/2	100	100	100	100
P-LATS T1097	100	108	107	115#
P-LATS S909	100	106	106	111
YAP	100	99	99	99
P-YAP S109	100	102	104	103
P-YAP S127	100	105	105	117#
P-YAP S397	100	112	113#	115#
TAZ	100	100	100	99
P-TAZ S89	100	110	106	115#
ERK2	100	100	100	99

	VEH	602	BTZ	602+B
PD-L1	100	90	96	86*
PD-L2	100	100	100	100
MHCA	100	111	111	120#
ODC	100	100	101	99
IDO1	100	100	100	96
p38 MAPK	100	99	100	99
P-p38	100	103	111	113#
BCL-XL	100	93	100	84*
MCL1	100	94	97	93
BAX	100	98	99	100
BAK	100	100	102	106
BIM	100	102	101	102
FLIP-s	100	98	102	102
ERK2	100	100	99	100

Supplementary Figure 3: (A) GZ17-6.02 and bortezomib regulate signaling pathways in U266 cells. Cells were treated with vehicle control, GZ17-6.02 (curcumin, 2 μM), bortezomib (10 nM) or the drugs in combination for 4 h. Cells were centrifuged and fixed *in situ*, permeabilized, stained with the indicated validated primary antibodies and imaged with secondary antibodies carrying red- and green-fluorescent tags. The staining intensity of at least 100 cells per well/condition is determined in three separate studies. The data are the normalized amount of fluorescence set at 100% comparing intensity values for vehicle control ($n = 3$ +/− SD). $^{\#}p < 0.05$ greater than vehicle control; $^{*}p < 0.05$ less than vehicle control. (**B**) GZ17-6.02 and bortezomib regulate signaling pathways in U266 cells. Cells were treated with vehicle control, GZ17-6.02 (curcumin, 2 μM), bortezomib (10 nM) or the drugs in combination for 4 h. Cells were centrifuged and fixed *in situ*, permeabilized, stained with the indicated validated primary antibodies and imaged with secondary antibodies carrying red- and green-fluorescent tags. The staining intensity of at least 100 cells per well/condition is determined in three separate studies. The data are the normalized amount of fluorescence set at 100% comparing intensity values for vehicle control ($n = 3$ +/− SD). $^{\#}p < 0.05$ greater than vehicle control; $^{*}p < 0.05$ less than vehicle control.

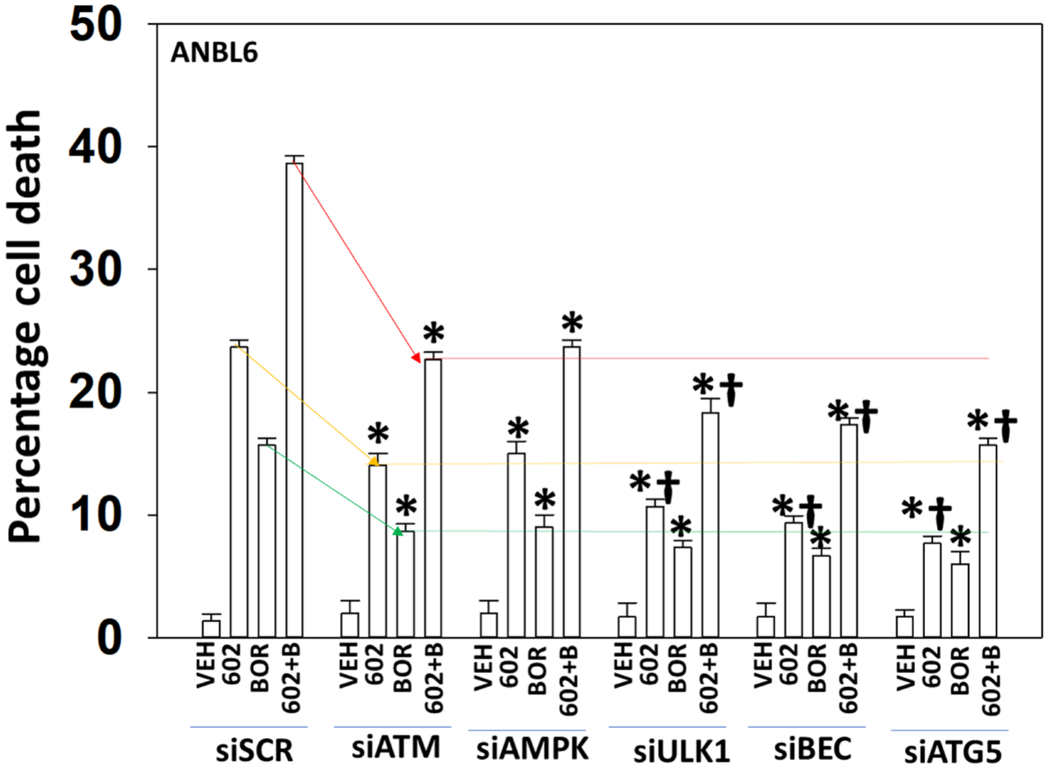

Supplementary Figure 4: Signaling by ATM-AMPK and autophagosome formation are toxic events when ANBL6 cells are treated with GZ17-6.02 and bortezomib. ANBL6 cells were transfected with a scrambled siRNA or with siRNA molecules to knock down the expression of ATM, AMPKα, ULK1, Beclin1 or ATG5. Twenty-four h later, cells were treated with vehicle control, GZ17-6.02 (curcumin 2 µM, final), bortezomib (10 nM) or the drugs in combination for 24 h. Twenty-four h later, cells were isolated, and viability determined via trypan blue exclusion assays ($n = 3$ +/− SD). $^{*}p < 0.05$ less than corresponding value in siSCR cells; $^{\dagger}p < 0.05$ less than corresponding values in siATM and siAMPK transfected cells.

	siSCR HDAC1 VEH	siSCR HDAC1 602	siSCR HDAC2 VEH	siSCR HDAC2 602	siSCR HDAC3 VEH	siSCR HDAC3 602	siBeclin1 HDAC1 VEH	siBeclin1 HDAC1 602	siBeclin1 HDAC2 VEH	siBeclin1 HDAC2 602	siBeclin1 HDAC3 VEH	siBeclin1 HDAC3 602
ALMC1	100	76*	100	70*	100	68*	100	97	100	92	100	90
ANBL6	100	80*	100	82*	100	79*	100	95	100	96	100	91
U266	100	80*	100	83*	100	84*	100	98	100	98	100	97

Supplementary Figure 5: GZ17-6.02-induced degradation of HDACs1/2/3 requires autophagosome formation. ALMC1, ANBL6 and U266 cells were transfected with a scrambled siRNA control or with an siRNA to knock down expression of Beclin1. Twenty-four h later, cells were treated with vehicle control or with GZ17-6.02 (2 µM curcumin, final) for 4 h. Cells were centrifuged and fixed *in situ*, permeabilized, stained with the indicated validated primary antibodies and imaged with secondary antibodies carrying red- and green-fluorescent tags. The staining intensity of at least 100 cells per well/condition is determined in three separate studies. The data are the normalized amount of fluorescence set at 100% comparing intensity values for vehicle control ($n = 3$ +/− SD). $^{*}p < 0.05$ less than vehicle control.

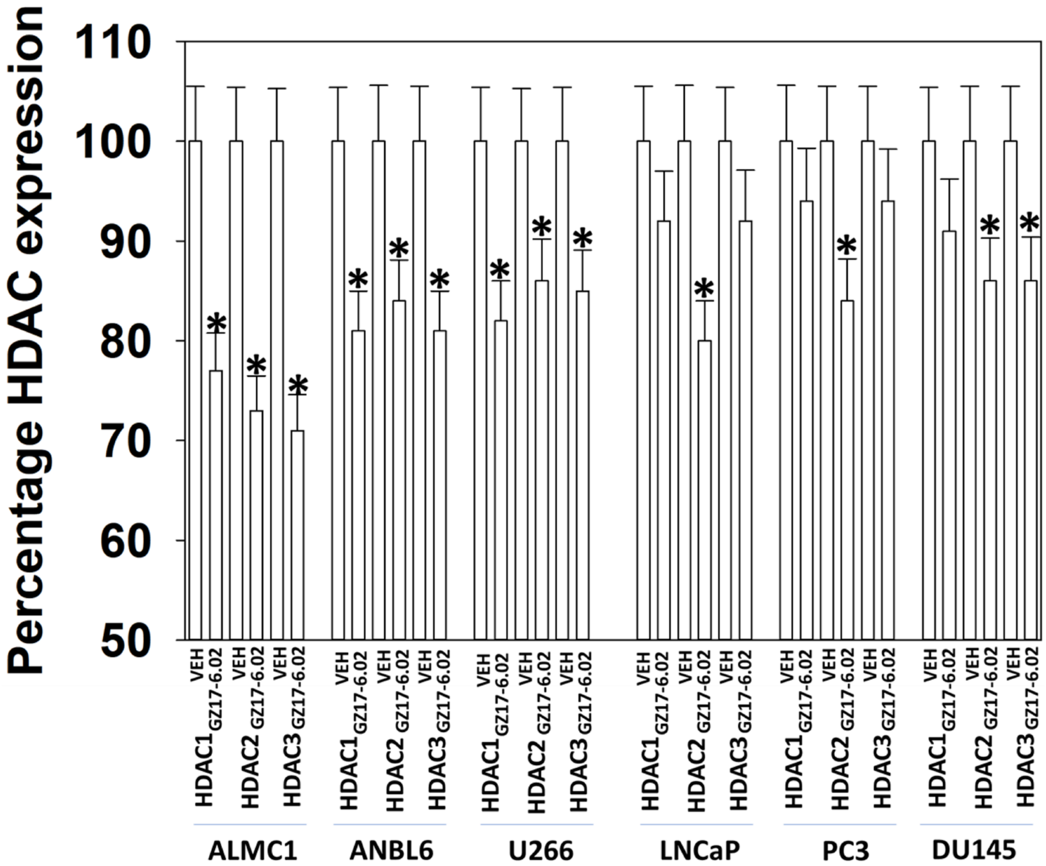

Supplementary Figure 6: GZ17-6.02 more effectively reduces the expression of HDACs1/2/3 in multiple myeloma cells than in prostate cancer cells. Multiple myeloma and prostate cancer cells were treated with vehicle control or with GZ17-6.02 (2 μM curcumin, final). After 4 h, cells were centrifuged and fixed *in situ*, permeabilized, stained with the indicated validated primary antibodies and imaged with secondary antibodies carrying red- and green-fluorescent tags. The staining intensity of at least 100 cells per well/condition is determined in three separate studies. The data are the normalized amount of fluorescence set at 100% comparing intensity values for vehicle control ($n = 3$ +/− SD). $^{*}p < 0.05$ less than vehicle control.

ALMC1

	4h VEH	4h 602	4h BTZ	4h 602+B	24h VEH	24h 602	24h BTZ	24h 602+B	48h VEH	48h 602	48h BTZ	48h 602+B
P-H3 T3	100	120#	104	121#	100	114#	105	116#	100	113#	107	114#
P-H3 S10	100	114#	105	121#	100	107	100	108	100	104	100	105
P-H3 T11	100	104	103	106	100	105	103	107	100	101	100	104
P-H3 S28	100	105	101	108	100	114#	103	115#	100	107	99	105
Histone H3	100	100	100	100	100	101	101	101	100	100	100	101

ANBL6

	4h VEH	4h 602	4h BTZ	4h 602+B	24h VEH	24h 602	24h BTZ	24h 602+B	48h VEH	48h 602	48h BTZ	48h 602+B
P-H3 T3	100	115#	105	118#	100	112	102	114#	100	110	107	113#
P-H3 S10	100	110	105	116#	100	113#	108	116#	100	109	100	113#
P-H3 T11	100	102	103	105	100	104	101	105	100	100	101	100
P-H3 S28	100	106	100	109	100	110	100	112	100	100	101	100
Histone H3	100	100	100	100	100	99	100	100	100	101	101	101

Supplementary Figure 7: GZ17-6.02 and bortezomib regulate histone H3 phosphorylation. ALMC1 and ANBL6 cells were treated with vehicle control, GZ17-6.02 (2 µM curcumin, final), bortezomib (10 nM) for 4 h, 24 h and 48 h. Cells were centrifuged and fixed *in situ*, permeabilized, stained with the indicated validated primary antibodies and imaged with secondary antibodies carrying red- and green-fluorescent tags. The staining intensity of at least 100 cells per well/condition is determined in three separate studies. The data are the normalized amount of fluorescence set at 100% comparing intensity values for vehicle control ($n = 3$ +/− SD). $^{*}p < 0.05$ less than vehicle control; $^{\#}p < 0.05$ greater than vehicle control.

ALMC1

	siSCR	siProtein
ATM	100	21
ERK2	100	100
AMPKα	100	29
ERK2	100	100
ULK1	100	24
ERK2	100	99
Beclin1	100	23
ERK2	100	99
ATG5	100	18
ERK2	100	100
HDAC1	100	22
ERK2	100	99
HDAC2	100	30
ERK2	100	100
HDAC3	100	25
ERK2	100	100

ANBL6

	siSCR	siProtein
ATM	100	30
ERK2	100	100
AMPKα	100	27
ERK2	100	100
ULK1	100	25
ERK2	100	101
Beclin1	100	26
ERK2	100	101
ATG5	100	22
ERK2	100	101
eIF2α	100	24
ERK2	100	100
BAK	100	26
ERK2	100	100
BIM	100	29
ERK2	100	99
CD95	100	28
ERK2	100	99
HDAC1	100	28
ERK2	100	100
HDAC2	100	30
ERK2	100	100
HDAC3	100	26
ERK2	100	100

Supplementary Figure 8: Control data showing siRNA knock down. ALMC1 cells as indicated were transfected with siRNA molecules to knock down the expression of the indicated proteins. The percentage remaining after knock-down is presented. (n = 3 +/− SD) (total ERK2 is included as an invariant total protein loading control).
